# A Crevice Corrosion Model for Biomedical Trunnion Geometries and Surfaces Feature

**DOI:** 10.3390/ma14041005

**Published:** 2021-02-20

**Authors:** Angela Bermúdez-Castañeda, Anna Igual-Muñoz, Stefano Mischler

**Affiliations:** 1 Tribology and Interfacial Chemistry Group, EPFL, École Polytechnique Fédérale de Lausanne, 1015 Lausanne, Switzerland; anna.igualmunoz@epfl.ch (A.I.-M.); stefano.mischler@epfl.ch (S.M.); 2 Research Group in Sustainable Design in Mechanical Engineering, DSIM, Escuela Colombiana de Ingeniería Julio Garavito, 111166 Bogotá, Colombia

**Keywords:** crevice-corrosion, modular implants, biomedical alloys, corrosion modelling

## Abstract

Modular hip joint implants were introduced in arthroplasty medical procedures because they facilitate the tailoring of patients’ anatomy, the use of different materials in one single configuration, as well as medical revision. However, in certain cases, such prostheses may undergo deterioration at the head–neck junctions with negative clinical consequences. Crevice-corrosion is commonly invoked as one of the degradation mechanisms acting at those junctions despite biomedical alloys such as Ti6Al4V and CoCr being considered generally resistant to this form of corrosion. To verify the occurrence of crevice corrosion in modular hip joint junctions, laboratory crevice-corrosion tests were conducted in this work under hip joint-relevant conditions, i.e., using similar convergent crevice geometries, materials (Ti6Al4V and CoCr alloys vs. ceramic), surface finish, NaCl solution pHs (5.6 and 2.3), and electrochemical conditions. A theoretical model was also developed to describe crevice-corrosion considering relevant geometrical and electrochemical parameters. To verify the model, a FeCr alloy, known to be sensitive to this phenomenon, was subjected to the crevice-corrosion test in sulfuric acid. The experiments and the model predictions clearly showed that, in principle, crevice corrosion of Ti6Al4V or CoCr is not supposed to occur in typical crevices formed at the stem-neck junction of hip implants.

## 1. Introduction

Hip joint prostheses are implants used to replace failed natural joints. Modern modular hip joints typically consist of a ball inserted on a stem through a conical junction with the trunnion on the top of the stem and the corresponding cone bore on the ball. Modular implants facilitate medical revisions and improve the prosthesis adaptation to the patient’s anatomy [1]. This modularity also allows the combination of different materials’ properties in one single configuration. Typically, the ball is made out of ceramics or CoCrMo alloys while titanium or other metals (stainless steels, CoCrMo alloys) are used for the stem [2]. Both metals exhibit the necessary corrosion resistance to withstand the contact with the synovial fluid, essentially a water solution containing organic molecules and ions such as chlorides with a pH around 7. Despite these advantages, modular hip joints have been associated with health complications such as adverse tissue reactions and high-metallic-ion levels in the blood [1,3,4]. These complications were attributed to the material degradation in the trunnion-bore contact by mechanisms such as fretting-corrosion and crevice corrosion [3,5,6,7,8,9].

Crevice corrosion is a form of localized corrosion encountered in metallic structures presenting cavities or recessed areas. In the recessed area, the refreshing of the solution is slow because of the geometrical confinement. As a consequence, the solution in the crevice undergoes corrosion-induced compositional changes with respect to the outside, continually refreshed area. Compositional changes include acidification, concentration of aggressive ions, and depletion of oxidizing agents (typically dissolved molecular oxygen) [10,11,12,13]. The reduction in oxygen concentration may shift the corrosion potential in the crevice toward lower values, generating an electrochemical cell between the inside and outside of the crevice (aeration cell) [10].

In the case of passive metals, such as most biomedical alloys, the corrosion potential in the crevice can locally shift to the active domain and thus generate, usually at a certain distance from the crevice mouth, high corrosion rates. This form of crevice corrosion can provoke significant material wastage in the trunnion-bore contact, leading to its loosening and to a mechanical instability of the implant with negative clinical consequences. However, the establishment of an active dissolution domain within the crevice can occur only under specific electrochemical conditions essentially dictated by the potential distribution inside of the crevice due to the ohmic (IR) drop in the electrolyte as described by Pickering et al. [14] and Valdes [15]. The IR drop depends on the crevice geometry, solution conductivity, and electrochemical properties of the metal [11,13,15,16,17,18,19,20].

Fretting corrosion occurs when a metallic contact exposed to an aggressive environment undergoes micro-motions, therefore making the contacting materials experience elastic deformation and, by sufficient micro-motion amplitude, rubbing [21,22,23,24]. Rubbing may remove the passive film from the contacting metal, thus exposing bare metal to the corrosive environment and exacerbating corrosion [25]. Under appropriate electrochemical conditions, the passive film rebuilds on the exposed areas and, therefore, passive conditions re-establish when rubbing ceases [26].

In the biomedical literature, both mechanisms were invoked to explain the appearance of sometimes severe degradation of the trunnion-bore contacts made of titanium or cobalt chromium alloys in hip implants [3,5,6,7,8,9]. Crevice corrosion was proposed as potential degradation mechanisms because of the crevice formed by the mismatch angle existing between the bore and the trunnion. Surprisingly, in the corrosion literature, crevice corrosion of titanium or CoCrMo alloys was reported to occur only in very extreme conditions such as temperatures higher than 100 °C or combinations of temperature and highly concentrated acid solutions (low pH) [12,27,28,29,30,31]. The appearance of crevice corrosion in trunnion contacts of hip implants is therefore questionable. Note that the corrosion products attributed in the literature to crevice effects can also be explained solely by fretting corrosion phenomena that are likely to occur in such contacts in the case of insufficient geometrical compliance or assembling force and of surface contamination prior to assembly [32,33,34]. Clearly, the possible occurrence of crevice corrosion in typical trunnion-bore contacts needs to be assessed.

The goal of this paper is to evaluate whether crevice corrosion of biomedical-grade titanium and cobalt-chromium can occur in typical trunnion-bore hip implant contacts. For this, electrochemical crevice corrosion experiments will be conducted using sample assemblies mimicking the typical conditions found in hip implants. The crevices are established between an inert ceramic bore and either Ti6Al4V or CoCrMo cones.

The experiments will be supported by a simple theoretical model predicting the ohmic resistance inside of the crevice and the corresponding drop in electrode potential. This model is based on Pickering’s original model [18] for constant-gap, smooth-surface crevices but was adapted to take into account the trunnion conical geometry, as well as surface roughness, together with the electrochemical properties of the alloys.

## 2. Materials and Methods

### 2.1. Materials and Electrolytes

The materials tested in this work were two biomedical alloys (CoCr and Ti6Al4V) and one reference FeCr alloy containing 15 wt.% Cr as the AISI430 stainless steel known to be susceptible to crevice corrosion [35]. The CoCr alloy was a low-carbon CoCrMo alloy containing 28 wt.% Cr, 6 wt.% Mo, 0.05 wt.% C, and 66 wt.% Co [36]. The Ti6Al4V grade 5 alloy chemical composition was 92 wt.% Ti, 5.6 wt.% Al, and 3.7 wt.% V [37]. Three different electrolytes were used for the electrochemical tests, as shown Table 1.

**Figure 1:**
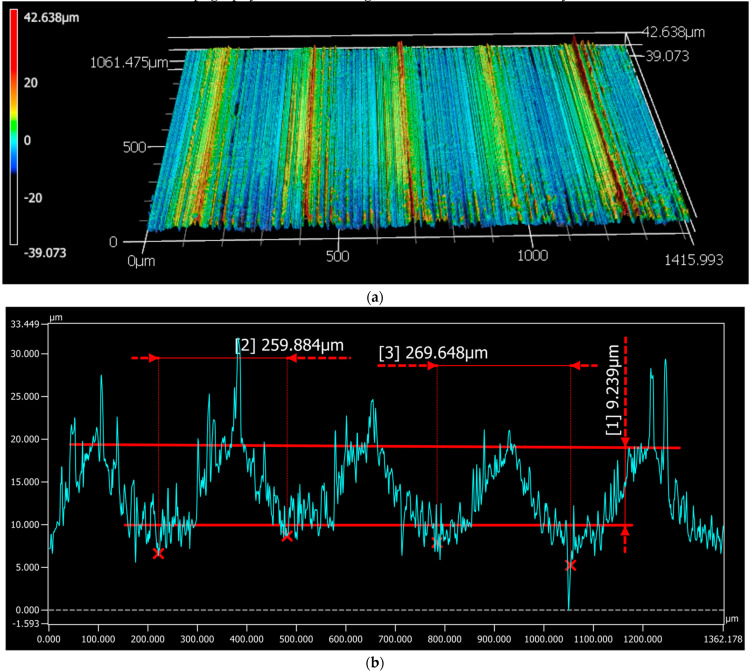
(**a**) 3D image and (**b**) line profile describing the surface topography of the metallic trunnions.

All alloys were fine machined in a controlled way in order to achieve a surface finish mimicking the patterns (thread and roughness) of real biomedical trunnions [38], as shown in Figure 1. 3D maps were performed using a laser scanning confocal microscope, Keyence VK-X200 Series 3D (Itasca, IL 60143, USA). Data analysis to determine the surface topography was done through the software MultiFileAnalyzer 1.3.0.115.

### 2.2. Corrosion Tests

Potentiodynamic curves of all alloys were carried out using an Autolab PGSTAT30 potentiostat (using the software NOVA) in a three-electrode configuration cell, with the alloy as the working electrode (WE), a platinum wire as the counter electrode (CE), and a saturated Ag/AgCl reference electrode (RE). All potentials will be reported with respect to the Ag/AgCl reference electrode (205 mV with respect to the standard hydrogen electrode, SHE). The potentials were swept at a rate of 2 mV/s from −1.5 to 1.5 V for the CoCr alloy; from −2 to 2 V for the Ti6Al4V; and from −1.2 to 1.5 V for the FeCr alloy.

### 2.3. Experimental Setup for the Crevice Corrosion Experiments

**Figure 2:**
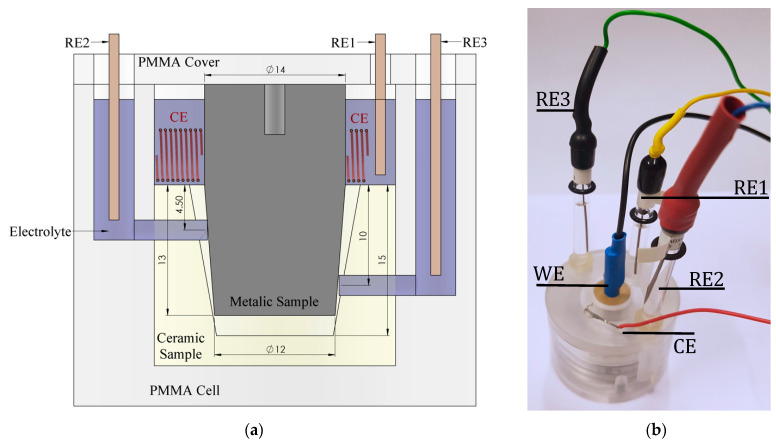
Cross section (**a**) and picture (all measurements are in mm) (**b**) of the electrochemical crevice setup.

The crevice corrosion setup developed for this study consists of a metallic working electrode (Figure 2a) machined as a trunnion and inserted in a bore made out of MACOR^®^ (Woodside, NY, USA), a machinable glass-ceramic. This configuration allows for creating a certain gap between the trunnion and the ceramic bore due to the angle mismatch between both components. The mismatch angle between the taper and the trunnion was 0.02°, which generates an aperture at the crevice mouth of 4.6 μm. These pieces were assembled by manually impacting a 500 g hammer with full arm momentum. The assembled components were placed in a PMMA electrochemical cell (Figure 2b) together with a platinum wire positioned around the taper outside of the crevice acting as the counter-electrode (CE) and three Ag/AgCl reference electrodes (REi). The reference electrodes were placed in three different positions along the trunnion in order to apply a selected potential and to register the potentials at different heights of the trunnion. RE1 was used to apply the selected potential and placed in the upper part of the cell (outside the gap). RE2 and RE3 were positioned inside the crevice at 4.5 and 10 mm, respectively, from the mouth of the gap (Figure 2a).

A total number of eight cells was used in parallel. Each cell was controlled by an AMEL 549 potentiostat used to carry out potentiostatic tests at different applied passive potentials. Two replicas of the tests under the same conditions were carried out to check for reproducibility. The area inside the cone was 2.5 cm^2^, while the material outside the crevice had a surface area of 3.1 cm^2^.

The response in current and the potential of the WE with respect to the different reference electrodes were monitored using a NI-6031E cardboard from National Instruments (32 channels in differential, resolution of 16 bits, a maximum frequency of 100 kS/s, and input range of +0.5 V) and a LabView program (version 5.1). A profilometer UBM Messtechnik, GMBH (Ettlingen, Germany), was used to measure the depth of the crevice formed during the experiments.

## 3. Results

### 3.1. Electrochemical Behavior of the FeCr, CoCr, and Titanium Alloys

**Figure 3:**
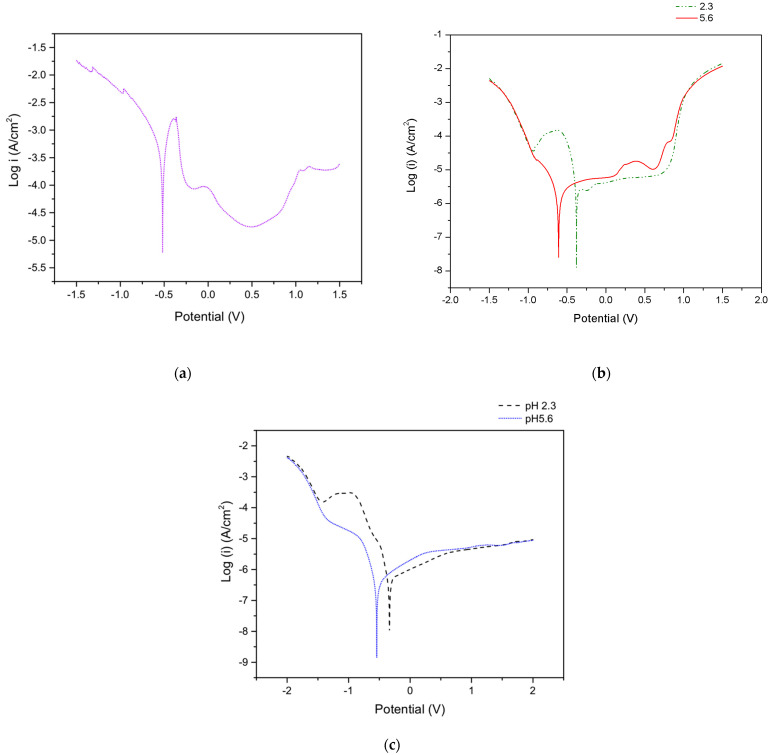
(**a**) Potentiodynamic curve of FeCr in 0.05 H_2_SO_4_, (**b**) CoCr in NaCl at pH 2.3 and 5.6, and (**c**) Ti6Al4V in NaCl at pH 2.3 and 5.6.

Polarization curves of FeCr, CoCr, and Ti6Al4V alloys are shown in Figure 3a–c, respectively. All materials show a passive plateau, but a clear active-passive domain is only observed in the FeCr curve after the anodic peak around −0.4 V.

Those curves were used to choose the potentials to be applied in the crevice corrosion experiments. For the FeCr, the potential was fixed at 0.5 V, which corresponds to the passive zone. For the CoCr, two distinct passive potentials were selected for the two solutions: −0.1 and 0.5 V for the pH 2.3 solution and −0.1 V for the pH 5.6 solution. For the Ti6Al4V, the selected passive potentials were 0.3 (pH 2.3 solution) and 0.5 V (pH 5.6 solution).

### 3.2. Crevice Corrosion Experiments

**Figure 4:**
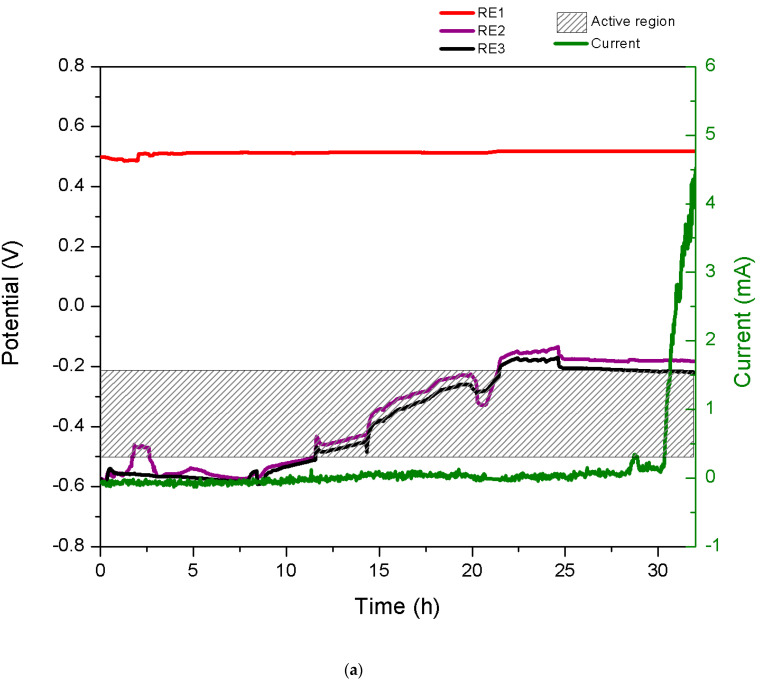

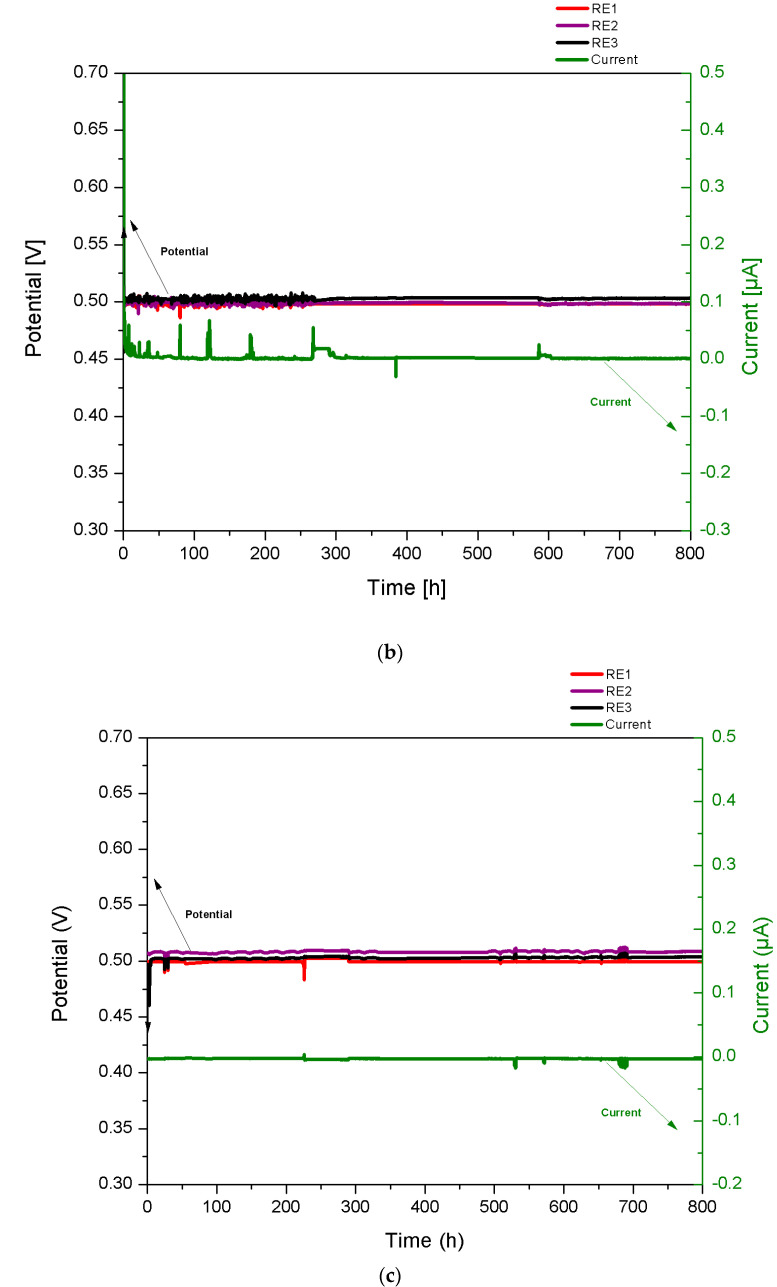
Time evolution of potential (in the two different positions of the trunnion, with respect to RE2 and RE3) and current of (**a**) FeCr in 0.5 H_2_SO_4_, (**b**), CoCr in NaCl pH 2.3, and (**c**) titanium in NaCl pH 5.6 at an applied passive potential of 0.5 V. In Figure 4a, the shadowed area corresponds to the potential region of the active dissolution.

Figure 4 shows an example of potentiostatic tests at applied passive potentials of 0.5 V carried out with the crevice-corrosion experimental setup including the current evolution with time and the potentials at different positions of the FeCr, CoCr, and Ti6Al4V trunnions in different solutions. In the case of the FeCr alloy, it is noticeable that the potentials measured at the beginning inside of the crevice (RE2 and RE3) are very low and even below the corrosion potential (−0.5 V) observed in the polarization curve shown in Figure 3a. This is probably due to the large ohmic drop established in the crevice that shifts the potential to significantly lower values. Later, the potential evolves to higher values with time.

The reason for that is not clear but is most likely caused by the increase in the pH inside of the crevice due to the consumption of the hydronium ions by the corrosion reaction. Indeed, the amount of the hydronium ions in the crevice can be estimated to be 5 × 10^−7^ mol by multiplying the volume of the crevice (approximately 5 × 10^−3^ cm^3^) by the hydronium concentration (0.1 M considering double deprotonating sulfuric acid). At the corrosion potential (−0.5 V), the crevice experiences a corrosion rate of approx. 0.5 mA/cm^2^ as determined by Tafel plot extrapolation from Figure 3a. By applying Faraday’s law, one obtains a corresponding reduction rate of the proton of 5 × 10^−9^ mol/s cm^2^. Considering the crevice surface area (5 cm^2^), this yields a hydronium ion reduction rate of 2.5 × 10^−8^ mol/s, which implies that the hydronium ion amount in the crevice (5 × 10^−7^ mol) is consumed by corrosion in the first 20 s. This confirms that the subsequent evolution of potential is likely controlled by the change in pH within the crevice. The current in Figure 4a remains very low until a sudden rise appears after 30 h of immersion, indicating that active corrosion starts taking place. Such an incubation period is commonly observed in crevice corrosion experiments [39].

The CoCr and Ti6Al4V alloys in Figure 4 show a very different behavior. The potentials inside of the crevice do not deviate from the imposed one even after 30 days of immersion. The current remains close to zero. This indicates that the crevice remains passive and no crevice corrosion occurs.

All systems were evaluated in duplicate and no significant differences in the behavior under crevice corrosion conditions were observed.

**Figure 5:**
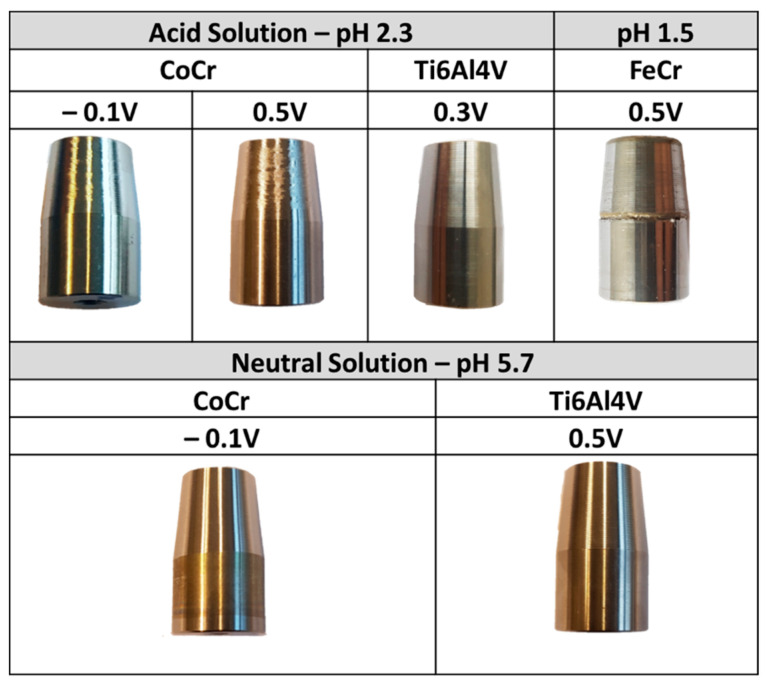
Pictures of the crevices of CoCr, Ti, and FeCr cones, after 1 month under crevice condition except for FeCr samples (2 days).

At the end of the crevice corrosion experiments (30 days for Ti6Al4V and CoCr alloys, 1.25 days for the FeCr alloy), the surfaces of the trunnion were characterized by optical and confocal microscopy in order to identify any corrosion damage. Figure 5 shows the final state of all tested samples.

**Figure 6:**
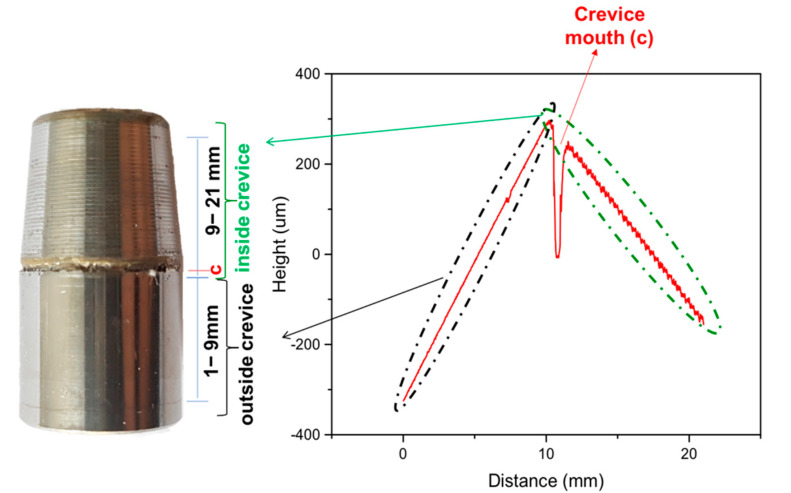
Surface profile measured using a UBM Telefokus scanning laser profilometer. Crevice corrosion in FeCr 15% taper evaluated during 2 days under crevice-corrosion conditions.

Figure 5 clearly indicates that crevice corrosion occurred only in the case of the FeCr alloy while CoCr and Ti6Al4V alloys did not exhibit any visible damage or sign of corrosion. In order to locate exactly the position of the crevice found in the FeCr sample, a surface profile was measured using the UBM Telefokus profilometer (Karlsruhe, Germany) (Figure 6). This allowed us to establish that the center of the crevice was located at 2 mm from its mouth.

## 4. Crevice-Corrosion Model

### 4.1. Geometrical Model

**Figure 7:**
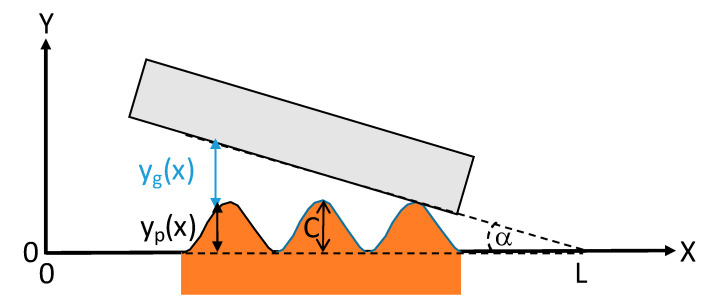
Sketch showing the determination of the crevice gap, $y_{g}\left(x\right)$, according to Equation (1).

The gap in the crevice was determined by considering the real surface profile of the metallic component. The cone profile (Figure 1) shows a peak-to-peak periodicity of 270 µm. For developing the numerical model, the surface profile over the entire crevice length was defined as a repetition of the experimental profile measured over a distance of 270 µm. To minimize the effect of experimental errors, the repeated pattern was taken as the average of three different 270 µm periods from the measured cone profile (all periods starting from their highest value). The obtained profile was then normalized so that the average height (central line average) is equal to zero. This cone profile will be repeated along the whole cone length. The real gap in the crevice, Figure 7, as a function of the distance from the crevice mouth entry ($y_{g}\left(x\right)$) was calculated by considering Equation (1).

(1) $$y_{g}\left(x\right)=\tan\alpha \left(L-x\right)+C-y_{p}\left(x\right)$$

where *α* is the angle between the cone and the head (0.02°), *C* is the maximum value of the input surface profile, *L* is the crevice length (13 mm), and $y_{p}\left(x\right)$ is the input surface profile.

### 4.2. Electrolyte Resistance in the Trunnion Crevice

The total resistance *(R(x))* inside the gap at different distances from the crevice mouth is calculated by summing the individual resistances *(R_i_(x))* in the different sections of the gap. The section is defined by the distance between two points of the experimental surface profile (Δ*x*).

The value of each section resistance, *R_i_(x)*, is obtained by multiplying the resistivity of the solution (ρ = 350 Ω mm) by Δ*x* and dividing by the surface area of the circular crown defined by the inner and the outer cone. The former is determined by its inner radius *(r_int_* = 6 mm) and the latter is calculated with the gap profile ($r_{ext}=r_{int}+y_{g}\left(x\right)$). Thus, the total resistance at a distance x from the crevice mouth entry, *R(x)*, is given by Equation (2).

(2) $$R\left(x\right)=\overset{L}{\underset{i=0}{\sum }}R_{i}\left(x\right)=\overset{L}{\underset{i=0}{\sum }}\rho \frac{\Delta x}{\pi {\left(r_{int}+y_{g}\left(x\right)\right)}^{2}-\pi {\left(r_{int}\right)}^{2}}$$

### 4.3. Electrochemical Conditions for Sustaining Crevice Corrosion in the Trunnion

Crevice corrosion results from the establishment of a stable electrochemical cell between the actively corroding inner surface of the crevice (anode) and the outer passive metal surface (cathode). In the case of taper trunnion junctions of hip joints, the depassivation of the originally passive inner surface can arise from the classical mechanism of oxygen depletion [10] or, more likely, from abrasion of the passive film during fretting [40,41].

To sustain crevice corrosion, it is necessary that the inner surface cannot repassivate. Repassivation occurs when two conditions are satisfied on each section of the crevice: Each area of the crevice has to reach the current density corresponding to the passivation current density, *i_pass_*, while keeping the potential above the passivation potential, *E_pass_* [14,16].

Assuming that each segment of the crevice is experiencing a current equal to *I_pass_*, this current corresponds to the product of *i_pass_* by the area of the inner cylinder at each gap distance, Equation (3)

(3) $$I_{pass}\left(x\right)=i_{pass}2\pi r_{int}\left(x\right)$$

In this case, the potential inside the crevice is given by Equation (4).

(4) $$E\left(x\right)=E_{out}-I_{pass}\left(x\right)R\left(x\right)$$

where *E_out_* is the potential established or applied at the metal surface outside of the crevice.

Note that in this equation, *I_pass_* is considered a net anodic current, and thus, it is valid only when cathodic currents are negligible, i.e., for potentials higher than *E_corr_*.

According to this, crevice corrosion can be sustained only when *E(x)* is lower than *E_pass_*. Thus, beyond a precise control of the surface topography and of the crevice geometry (α, L, and *r_int_*), in order to assess the risk of crevice corrosion, it is important to determine the *E_pass_* and the *i_pass_* of the biomedical alloy.

### 4.4. Modelling Fe-Cr Behavior

**Figure 8:**
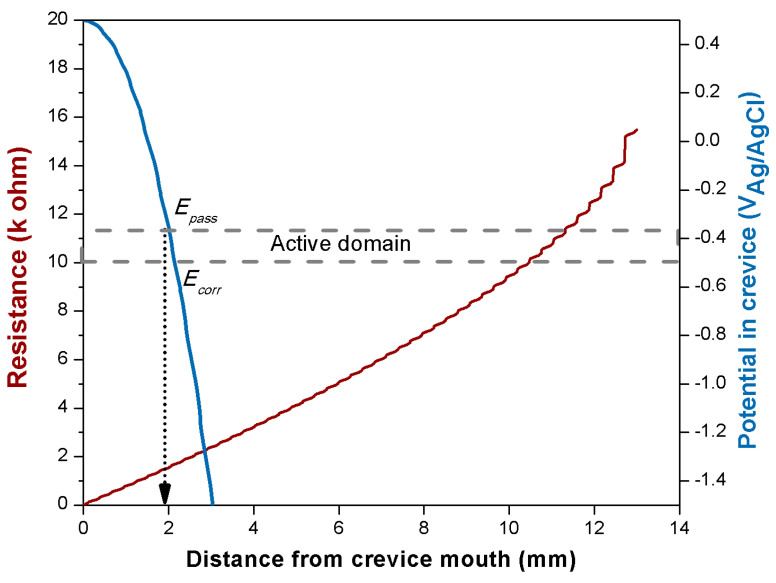
Resistance and potential evolution for the Fe−Cr trunnion inside the crevice as a function of the distance from the crevice mouth.

From the potentiodynamic curves of Fe-Cr in the H_2_SO_4_ solution, Figure 3, *E_pass_* and i_pass_ can be extracted and values of −0.37 V and 1.8 mA/cm^2^ were obtained, respectively. The applied passive potential *E_out_* is 0.5 V. Figure 8 shows the evolution of the resistance *R(x)* with distance, *x*, from the crevice mouth (Equation (2)) and the corresponding evolution of the potential, *E(x)*, inside the crevice (Equation (4)).

For comparison, the potential domain corresponding to the active dissolution and delimited by *E_corr_* and *E_pass_* is also shown. From this graph, it appears that Fe−Cr surfaces are passive only up to a depth of approximately 1.8 mm. Between this depth and the roughly 2 mm distance where the potential corresponds to *E_corr_*, the surface should corrode actively. Below 2 mm, the potential drops well below *E_corr_*, and thus, the Fe−Cr anodic reaction rate, i.e., the corrosion rate, becomes negligible.

In other words, this model predicts that corrosion of Fe−Cr in the present experimental crevice conditions should occur only at a distance of approximately 2 mm as indeed experimentally observed (see Figure 6). This validates the theoretical approach developed here.

### 4.5. Modeling Ti6Al4V Behavior

**Figure 9:**
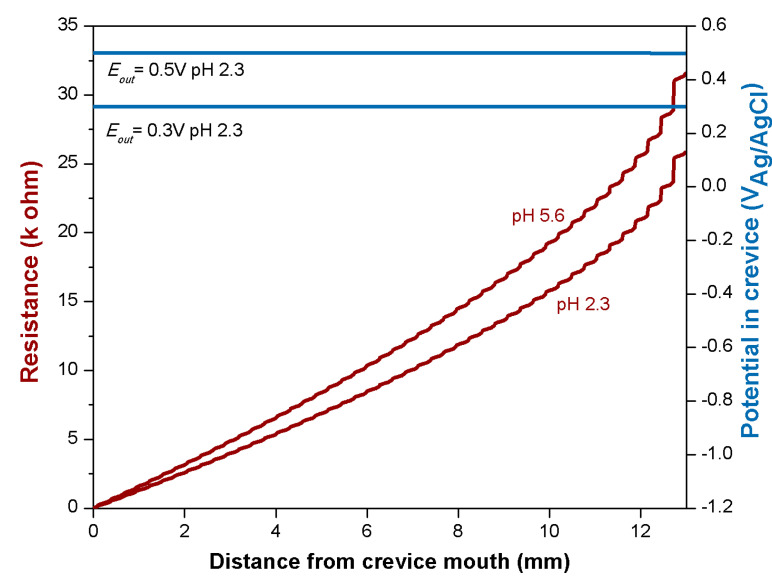
Resistance and potential evolution for the titanium trunnion inside the crevice as a function of the distance from the crevice mouth at different applied potentials (*E_out_*) and pHs.

The polarization curves measured on titanium, Figure 3, do not allow the identification of a distinct active-passive transition, and therefore, it is not possible to determine *E_pass_* and *i_pass_* from these measurements. In the literature, Kaesche [42] reported measurements of the potentiodynamic curves of titanium in different concentrations of H_2_SO_4_ ranging from 0.05 to 3 M. Those experiments show that the *i_pass_* rapidly decreases with the dilution of H_2_SO_4_. For a concentration of 0.25 M where an active peak can still be distinguished, the *i_pass_* is 0.01 µA/cm^2^. At higher pH, as of interest here, the *i_pass_* value of titanium is supposed to be significantly lower. However, for the calculations of the potential inside the crevice, Figure 9, the value of 0.01 µA/cm^2^ was taken as an upper limit. Due to this very low *i_pass_*, the calculated potential inside the crevice (Figure 9) does not show significant variations with respect to the applied one (*E_out_*) and this is at any distance from the crevice mouth. This is in good agreement with the experimental results obtained in the present work, Figure 4, where differences in potential between the different reference electrodes were very small (less than 50 mV).

Kaesche [42] also reported that the *E_pass_* of titanium slightly increases with acid concentration. At a concentration of 0.25 M H_2_SO_4_, the *E_pass_* is reported as being −0.75 V_Ag/AgCl_. At higher pH, the *E_pass_* is supposed to be even lower. According to the present calculations (Figure 9), such low potentials are never reached in the 13 mm long crevice of the titanium sample and, thus, crevice-corrosion is not sustainable under the present conditions. This indeed corresponds to the experimental observations where no signs of corrosion were observed.

### 4.6. Modelling CoCrMo Behavior

**Figure 10:**
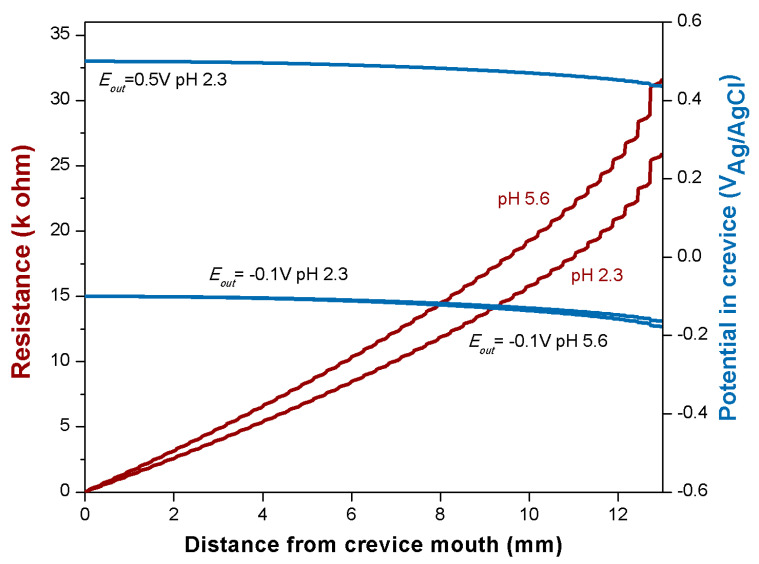
Resistance and potential evolution for the CoCrMo trunnion inside the crevice as a function of the distance from the crevice mouth at different applied potentials (*E_out_*) and pHs.

Similarly to the case of titanium, no active-passive transition can be distinguished in the polarization curves, Figure 10. Interestingly, to the best knowledge of the authors, such a transition was never reported in the literature. Some CoCrMo potentiodynamic curves measured in PBS exhibit a pseudo-active peak at −0.9 V_Ag/AgCl_ at the beginning of the anodic domain with a maximum current density around 1–2 µA/cm^2^, while others measured by different laboratories under identical conditions did not show such a feature [43]. The reasons for these discrepancies are not yet clear and generate doubts about the effective existence of active dissolution for these kinds of alloys. Indeed, active peaks were not observed in polarization curves measured in H_2_SO_4_ [44].

Süri [45] reported that an active peak on a CoCrMo alloy immersed in sulfuric acid was only observable when sulfides were added to the solution. All this suggests that the CoCrMo alloys are spontaneously passive in a wide range of environments. Nevertheless, for the present calculations, we consider the pseudo-active peak reported in the literature with *i_pass_* and *E_pass_* values of 2 µA/cm^2^ and −0.9 V_Ag/AgCl_, respectively. The calculation outcomes are shown in Figure 10. The potential evolutions inside the crevice for all considered pHs and applied potentials show a slight lowering of the potential (less than 100 mV) with distance from the crevice mouth. Nevertheless, the passivation potential remains more cathodic than the lowest calculated ones and, thus, the model predicts the absence of crevice-corrosion.

The experimental results are in partial agreement with the model predictions. Indeed, as predicted by the model, no crevice-corrosion could be detected in the tested CoCrMo samples (Figure 10). However, the monitored potential along the crevice does not deviate significantly from the imposed one (Figure 4) and thus does not correspond to the calculated one. Indeed, for the position of RE2 (4.5 mm from the crevice mouth), the calculated potential drop is very small, 20 mV, and less than 100 mV at 10 mm distance (position of RE3). This last decay was not experimentally observed. Note that the calculations are highly dependent on the considered value for *i_pass_* that, in the case of CoCrMo alloys, still remains ill-defined as discussed above. Assuming for the calculations an *i_pass_* value of 0.5 µA/cm^2^ already suppresses any significant variation in the calculated potential as a function of the distance from the crevice mouth, this is in agreement with the experimental results. This clearly shows the importance of disposing reliable data for the passivation current densities and the passivation potentials to assess the occurrence of crevice corrosion in hip implants.

## 5. Discussion

The theoretical approach proposed here to assess the occurrence of crevice corrosion in crevice geometries and sizes mimicking the trunnion/bore contact of hip joints correctly described the absence of crevice corrosion in the Ti6Al4V and CoCrMo alloys in sodium chloride, as well as the location of the crevice corrosion site in the case of the FeCr alloy.

The proposed model allows us to assess the role of electrochemical parameters (passivation potential *E_pass_* and passivation current density *i_pass_*), geometrical factors (mismatch angle, crevice length), and surface topography on the occurrence of crevice corrosion.

As mentioned above, the determination of the electrochemical parameters for metals such as Ti and CoCr alloys, which do not exhibit a clear active/passive transition in the polarization curves, is problematic. Dedicated studies using existing techniques (such as galvanostatic polarization curves [46]) or implying advanced corrosion techniques (such as Electrochemical Quartz Crystal Microbalance (EQCM) are needed to provide more reliable data for *E_pass_* and *i_pass_* as crucial parameters for the crevice corrosion.

**Figure 11:**
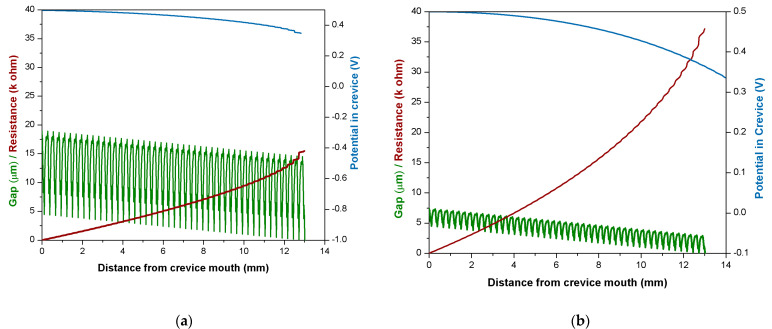
Calculated potential drops in the crevice for (**a**) the roughness profile shown in Figure 1 and (**b**) a smoother roughness profile with 5 times lower profile heights with respect to (**a**). The gap profile is shown in green.

Figure 11 shows the simulated effect of surface roughness on the crevice corrosion for a hypothetical metal, which exhibits a potential of 0.5 V and an *i_pass_* value of 2 × 10^−3^ mA/cm^2^. Two roughness profiles were compared: The original profile used in the previous calculations and mimicking the typical surface state of hip implant trunnions (Figure 11a) and the same profile but with 5 times lower profile heights (Figure 11b), i.e., a much smother surface. The smoothening of the surface clearly reduces the interstitial space left between the trunnion and bore, and thus, the electrolyte resistance and the associated drop in potential become larger, thus favoring in principle the appearance of crevice corrosion.

**Figure 12:**
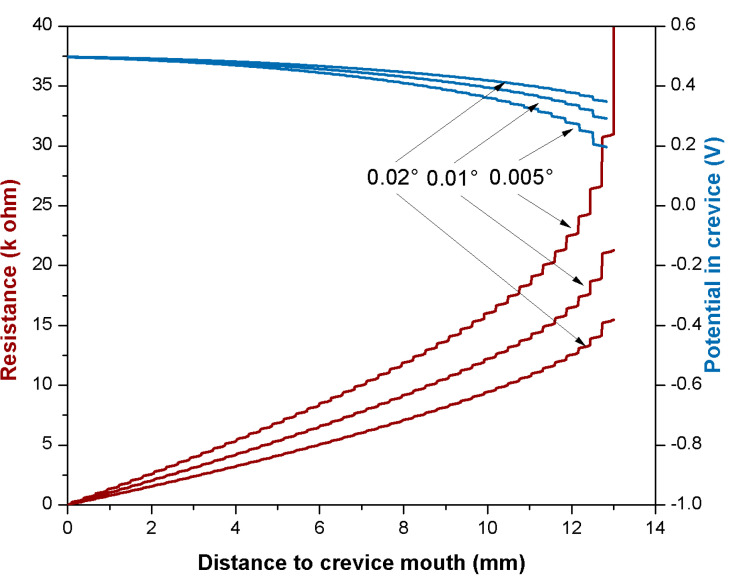
Calculated resistances and potential drops in the crevice for different mismatch angles.

The effect of the mismatch angle is modeled in Figure 12 for the same metal as in Figure 11. In this case, the mismatch angles of 0.02° (as in the experiment and the previous calculations), 0.01°, and 0.005° were considered. Lower mismatch angles imply narrower gaps and, therefore, as shown in Figure 12, higher electrolyte resistances and potential drops in the crevice and, therefore, a higher risk of crevice corrosion.

## 6. Conclusions

A laboratory experiment was designed for assessing the occurrence of crevice corrosion in geometries and surface finish conditions mimicking the metallic trunnion versus ceramic bore contact found in typical hip joint implants.

Experiments conducted at the imposed passive potential with an FeCr alloy in sulfuric acid and Ti6Al4V and CoCrMo biomedical alloys in saline solution at two different pHs showed that under these conditions, CoCrMo and Ti6Al4V alloys are immune of crevice corrosion at least for 30 days.

A crevice corrosion model based on Pickering’s electrochemical approach was developed to assess the potential distribution inside of the crevice due to the electrolyte electrical resistance. The model accounts for the convergent geometry of the gap between the conical trunnion and conical bore and for the specific surface topography of hip joint implants. In agreement with the experimental observations, the model predicts the occurrence of crevice corrosion in the FeCr alloy and its absence in the case of the biomedical alloys.

This model approach points out the relevance of crucial parameters affecting crevice corrosion. Relevant electrochemical parameters are the passivation potential and the passivation current density that need to be precisely determined. Moreover, the model shows that decreasing the mismatch angle between the trunnion and bore or the surface roughness of the trunnion enhances the risk of crevice corrosion.

## Figures and Tables

**Table 1:** Properties of the test solutions *.

Solution	pH	Conductivity (mS/cm)
H_2_SO_4_ 0.05M	1.5	28.3
NaCl 0.9 wt.%	2.3	17.1
NaCl 0.9 wt.%	5.6	14.0

* Reagents used to prepare the dissolutions were: Sulfuric acid 95–97% (MERCK, Zug, Switzeland), sodium chloride ACS reagent, ≥99.0% (Sigma Aldrich, Darmstadt, Germany), double distilled water.

## Data Availability

The data presented in this study are available in https://www.dropbox.com/sh/szb0rrsetke8equ/AABJXBw2l7nP6iVSw8VtOCUea?dl=0.
